# Intermittent Feeding Schedules—Behavioural Consequences and Potential Clinical Significance

**DOI:** 10.3390/nu6030985

**Published:** 2014-03-04

**Authors:** Michelle Murphy, Julian G. Mercer

**Affiliations:** Rowett Institute of Nutrition and Health, University of Aberdeen, Greenburn Road, Bucksburn, Aberdeen, AB21 9SB, UK; E-Mail: j.mercer@abdn.ac.uk

**Keywords:** binge eating, irregular feeding, meal schedules, anxiety, food anticipatory activity, behaviour

## Abstract

Food availability and associated sensory cues such as olfaction are known to trigger a range of hormonal and behavioural responses. When food availability is predictable these physiological and behavioural responses can become entrained to set times and occur in anticipation of food rather than being dependent on the food-related cues. Here we summarise the range of physiological and behavioural responses to food when the time of its availability is unpredictable, and consider the potential to manipulate feeding patterns for benefit in metabolic and mental health.

## 1. Introduction

Typically in modern society, eating behaviour is focused around meals, and the number and frequency of meals eaten is influenced by a number of factors, many of them cultural [1]. Here we will focus on the effects of meal frequency and timing on behaviour, in particular feeding and anxiety related behaviours, and their association with weight regulation, metabolic health and mood. Although some epidemiological studies suggest that the number of meals typically consumed per day may be inversely related to BMI, with increased meal number having a beneficial effect on body weight, the robustness of this relationship has been questioned [2]. Similarly, a systematic review of the relationship between eating behaviour (e.g., meal avoidance, irregular eating) and excess body weight concluded that this relationship was scarce or inconsistent [3]. In contrast, reducing food consumption by periods of intermittent fasting or extended interval between meals is thought to have some beneficial effects on health such as reduced risk of neurological or cardiovascular disease [4,5]. There is also the potential of meal intake at different times of day to differentially impact on cognitive performance and mood [6]. The design of public health interventions based around eating/food access schedules needs to be carefully considered; even apparently simple regimes designed to encourage healthy eating, such as restricting access to snack foods in children, may be undermined by increased positive feelings of palatability and desire directed to the unobtainable snack, and subsequent behavioural responses leading to an increased intake when the snack is again provided [7].

Feeding behaviours such as binge eating have been suggested to be the result of behavioural disinhibition following repeated periods of dieting or caloric restriction [8]. In line with this, motivation to obtain highly palatable food increases during dietary restriction, and even when animals are given free access to food, prior exposure to a repetitive cycle of intermittent access to palatable diet maintains a high level of compulsion-like motivation to eat sweet reward food despite negative reinforcement by foot shocks [9]. This would suggest that manipulating intervals between meals or feeding opportunities, and the ability to predict the reward value of food, may affect reward seeking behaviour as well as food consumption due to changes in individual restraint or inhibition. With this in mind, it is easy to envisage some form of relationship between over consumption of food and the use of addictive substances. Indeed it has been suggested, based on cross sensitisation studies and clinical observations, that occurrence of binge eating and other eating disorders are positively correlated with drug addictions [10,11,12,13]. A primary outcome in both areas of research, *i.e.*, eating disorders and addiction, is the development of anxiety-related behaviours, manifested in rodent models as behaviour in the elevated plus maze (EPM) [14,15]. This highlights the need for greater understanding of how eating patterns might affect anxiety and other mood related conditions. This evidence gap assumes greater significance in the light of the prevalence of mental disorders in the general population, which for the EU, for example, now exceeds one third of the population per year [16]. The difficulty here lies with the recognised lack of animal models that show appropriate characteristics of binge eating disorder [17]. Binge eating disorder can be sub-classified clinically based on the sequential association of dieting (food restriction) and binge eating (*i.e.*, binge-first or diet-first), but patients in both categories have similar psychiatric symptoms, with more than a third presenting with anxiety disorders and over 60% having some form of mood disorder [18]. Therefore investigating diets of variable composition and with manipulated access regimes may uncover potential animal models for binge eating disorder. The usefulness of such models would be increased if a relationship could be established with a robust behavioural test, such as those available for anxiety.

Another condition associated with the timing of meals is night eating. In humans this is characterised by the skipping of morning meals and the consumption of more than 50% of daily food intake in the evening, including appreciable intake after the evening meal, leading to metabolic consequences such as disruption of circadian patterns of melatonin, leptin, insulin and glucose [19,20]. These findings suggest that meal timing in humans may influence energy homeostasis and additional circadian-driven behaviours. Similar observations have been made in rodent models where shifting normal feeding time desynchronises circadian rhythms and results in metabolic disorders and weight gain [21]. In contrast, restricting food availability, rather than calorie intake, can have beneficial effects: mice fed an obesogenic high fat diet for only 8 h per day in the dark phase were protected against obesity and metabolic complications despite consuming equivalent calories [22]. Taken together, the above findings suggest that regulating eating pattern has the potential to impact on both mental and metabolic health. An improved evidence base in this area could lead to strategies to improve the health and well-being of individuals.

An increase in total activity is often an indicator of an anorexia-like phenotype in rodents [23], and intermittent access to food is not only associated with increased binge-like consumption but also with increased physical activity in a model of activity-regulated-anorexia. This further supports the influence of feeding patterns on subsequent eating disorders and associated behaviours. Furthermore, rats fed at unpredictable times lose more weight and have increased activity relative to animals fed at a fixed time [24]. Intermittent access to food following a schedule of repeated cycles of restriction and refeeding led not only to binge-like consumption of either chow or a palatable cookie/chow based diet, but also disrupted sensory-specific satiety. Consequently, rats ate more at a second meal of the same food than would otherwise be the case [25], Compromised sensory-specific satiety is also a characteristic of bulimia in humans [26].

Some of the characteristics of human food-related disorders including binge eating disorder, night eating syndrome, anorexia and bulimia can be replicated in animal models by manipulating the time of food availability. However, we still have only limited information about how such feeding schedules affect physiology and behaviour, whether disruption of feeding can lead to increased risk of such eating disorders, and whether feeding schedules could be manipulated to develop an optimum routine which prevents the development of, or aids the recover from, such conditions. Here we examine the varied feeding schedules employed with animal models. Food-related behaviour is compared and contrasted on the basis of whether the imposed meal schedules are predictable, and thereby allow entrainment, or otherwise, before the likely mechanistic basis of these behavioural responses is considered.

## 2. Body Weight and Metabolic Health Outcomes

In general, a typical schedule of 2 h of access to a highly palatable diet provided daily in a predictable manner, with chow diet available for the remaining 22 h, is not sufficient to cause an increased body weight [27,28]. Intriguingly, mice fed a palatable food under a time restricted protocol (one access period of 8 h) consumed the same number of calories across this period as *ad lib.* high fat fed animals [22], but were protected from weight gain. This may support the theory that frequency of feeding episodes rather than size of meals or total energy intake contributes most to weight gain and the development of obesity [29], but certainly places emphasis on the need to understand the mechanisms underlying meal eating, and the potential benefits of understanding these.

Despite the ability of animals to regulate weight on schedule fed palatable diets with chow available *ad lib.*, this regime may lead to a deteriorating metabolic state, since blood glucose levels immediately prior to a 2 h scheduled feed were elevated on high fat pellet diets and had similar trends for sucrose and Ensure liquid supplements [28]. Rats on a high fat diet provided *ad lib.* or in scheduled meals with chow available for the remainder of the light:dark cycle had impaired glucose tolerance regardless of access schedule [27]. However, restricting the feeding time of mice to 8 h, while allowing the same level of calorie consumption as in *ad lib.* fed animals, prevented high fat induced changes to insulin levels or glucose control [22].

There is very limited information available on the use of unpredictable meal schedules and their effect on basic elements of metabolism and energy regulation. While it has been suggested that randomising the time of a meal reduces body weight compared to a fixed feeding schedule [30], this has not been shown relative to food intake. Other studies using such schedules have similar limitations or have not reported this information and it is therefore not possible to draw comparisons between predictable and unpredictable schedules in relation to weight regulation and metabolic status at this point.

## 3. Food Related Behaviours

### 3.1. Predictable Meal Schedules

Food anticipatory activity (FAA), the elevated locomotor activity occurring 2 h prior to a predicted meal in laboratory rodents, is relatively well defined for a variety of similar dietary manipulations based around predictable meal feeding. FAA in response to scheduled food access, or limited availability of a more palatable alternative food, is a proxy for a range of behaviours that are temporally dynamic, as well as psychological and cognitive processes such as perception, motivation, memory and decision making [31]. These processes are all relevant to human interactions with the food environment, and to eating disorders.

Learning when food will be available, for example, in the studies described above, is likely to involve inputs from both light- and food-entrainable oscillators as well as interval timers tracking light/dark periods and inter-meal intervals [31]. Whereas the circadian signal generated by the suprachiasmatic nucleus (SCN) relates directly to the light/dark cycle, coordinating many timing events and clock cells throughout the body, FAA represents the integrated output of SCN-independent peripheral and central food-entrained oscillators (FEOs) synchronised by limited but predictable food availability (usually single daily meals). For some circadian processes, particularly peripheral rhythms, the timing of food intake may be more important in the establishment of appropriate circadian oscillation than the prevailing light/dark cycle [32]. By contrast, SCN rhythms are not affected by feeding schedule in a regular daily light/dark cycle. There are a number of components of this food-entrainable system which are poorly understood at present, including the food-related stimuli that entrain the oscillators, and the anatomical distribution of these structures. Current evidence suggests that FEOs involved in FAA are either extra-hypothalamic or exist as part of a network with considerable redundancy, in the hypothalamus or beyond [32].

Experimental feeding schedules where animals are fed *ad lib.* but provided with additional timed access to a palatable diet, such as a high fat pellet or chocolate, frequently induce increased total energy intake. For logistical simplicity, the palatable diet is usually provided at the same time each day, allowing the “treat” to be anticipated by the animal using environmental and biological cues. For example, a study that combined twice weekly caloric restriction with a refeeding schedule the following day that included 2 h access to a sweetened fat diet produced a substantial binge-like feeding episode [33]. This and similar feeding schedules have been utilised to investigate the causes and consequences of binge eating. Interestingly, binge-like feeding behaviour was maintained when rats were re-exposed to the restriction/scheduled access manipulation after 2 weeks back on *ad lib.* chow access [33].

Delivery of palatable chocolate treats at a fixed time of day to *ad lib.* fed rats entrains anticipatory activity, albeit at a lower level than in rats held on a 2 h restricted access chow schedule [34]. FAA was maintained for 4 days when chocolate was withheld, and during 3 days of complete starvation [34]. Similarly, mice habituated to 2 h scheduled access to high fat diet with chow diet available for the remaining 22 h also exhibited FAA prior to the scheduled access period and, furthermore, maintained both FAA and changes in feeding pattern during a 7 day withdrawal period when only chow was available [35]. In another study, rats on *ad lib.* chow with predictable access to chocolate for 2 h in the light phase increased their activity levels relative to those fed *ad lib.* chow, but not relative to rats on a predictable restricted chow schedule [36]. This confirms previous studies demonstrating that palatable food in the form of nutrient-rich mash was capable of entraining wheel running in *ad lib*. fed rats [37]. However, by contrast, in a study feeding liquid chocolate Ensure Plus for 2 h as a supplement to *ad lib*. fed chow, less than half the rats tested exhibited FAA, whereas rats fed Ensure only for just 2 h a day showed robust FAA beginning 2–3 h before scheduled feeding [38]. Similarly, male mice failed to show any food anticipatory wheel running for palatable Fruit Crunchies, chocolate chips or chocolate Slimfast when standard chow was also available *ad lib*. However, a slight increase in locomotor activity was detected in these mice in response to a high fat chow snack [39], and results suggested that FAA under these conditions may be a function of feeding location or exploration rather than running behaviour, with mice tending to spend increased time nosing the feeding location [39]. Interestingly, female mice, while showing no indication of FAA under the same conditions, had increased activity and spent more time at the feeding location upon withdrawal of the palatable diet despite continued access to chow [39]. This rodent behaviour may provide some equivalence to the higher susceptibility to both eating disorders [40] and addictive behaviours in female human populations. For example, methamphetamines affect intake and behaviour of females more markedly than that of males, with both rodent and human studies showing females have greater dependence on, intake of, and associated seeking behaviours for methamphetamine [41,42]. As is frequently the case, studies of female animals are under-represented in the rodent literature in this area.

Scheduled feeding of standard chow or food of increased palatability has not only been shown to induce binge-like consumption but to also impact on circadian patterns [21]. While activity levels in rodents usually follow a diurnal pattern there is evidence to suggest that FAA can interrupt this pattern of activity, and that the palatability and quantity of food provided may affect the occurrence and extent of locomotor activity preceding a meal [37,43]. In a study where mice were fed every second day, oxygen consumption was reduced both on days when food was provided and on fasting days [44]. Although not measured, this reduction in energy expenditure is likely to be associated with a reduction in feeding related activity, which might contribute to significant changes in energy balance should fasting and binging occur intermittently over a prolonged period of time.

In addition to effects on locomotor activity and energy balance, food restriction can influence consumption of, and response to, drugs of abuse. These relationships were shown to intertwine in a study examining the interaction between feeding schedules under restriction conditions and locomotor responses to methamphetamine. Mice were food restricted to one of two body weight levels (8% or 16% weight loss) and fed in either one or 3 meals [45]. At the lower level of weight loss, only the mice fed 3 meals showed locomotor sensitisation to methamphetamine. Cross-sensitisation has been suggested between palatable foods, especially sucrose provided on a scheduled access regime, and addictive substances including ethanol, amphetamines and cocaine [46,47,48]. Interestingly, the study by Gosnell also links this relationship to a cross-sensitisation in activity levels [48]. The compulsive nature of binge eating either on a palatable diet or promoted with a restricted access schedule clearly has resonance to intake of addictive substances, and lessons learned from feeding studies may therefore have implications in other fields.

Another shared characteristic of addiction and eating disorders is an increase in anxiety-related behaviours. The prevalences of a spectrum of anxiety-related conditions are positively correlated with eating disorders [49], and experimental manipulation of food availability may also influence anxiety. Food restriction which allowed 2 h access to food at the same time every day induced anxiolytic outcomes in rats exposed to the elevated plus maze, and these effects persisted for 10 days beyond the end of the period of restriction [50]. However, comparison of different levels of restriction (25% or 50% for 3 weeks) in open field or elevated plus maze tests did not reveal any obvious dose-dependency in the anxiolytic effect [51]. In a different model, intermittent access to a sugar solution or palatable diet caused binge-like feeding, and subsequent withdrawal of palatable diet induced symptoms of anxiety. These symptoms mirror those observed upon withdrawal from addictive substances and may include teeth chattering and reduced open arm exploration on the elevated plus maze, although this has not been assessed during periods of bingeing on the palatable solution [14,52,53]. These findings are suggestive of similarities between certain eating behaviours and addiction to substances of abuse. While circadian rhythmicity of temperature and activity can be affected by both food and drug rewards, it is likely that these processes are separately regulated, and by more than one oscillator, with two distinct rhythms being apparent especially during drug withdrawal [54]. Overall, it is evident that when laboratory rodents are fed either standard or palatable diet over a set period of time in a meal-like format, they are capable of predicting its arrival and indulging in binge-like consumption, associated with changes in activity, eating behaviour and anxiety. Whereas the main behaviour focused on to date has been activity, further research examining mood and anxiety-related behaviours would be beneficial, and would clarify the association between feeding schedules, eating disorders and addictions.

### 3.2. Unpredictable Meal Schedules

Unpredictable meal provision is less well studied than scheduled delivery, at least in part due to logistical considerations. While FAA is reliably induced under predictable feeding schedules, it was not evident when rats were fed a 2 h meal at an unpredictable time in the light phase, although there was increased activity in phase with the previous meal, as well as immediately following food delivery [30]. An investigation of FAA in mice across a range of circadian and non-circadian intervals, changing the number of meals per day and the interval between meals, suggested that FAA was greatest during the light phase, although the interval between meals appeared to have little impact [55]. However, as FAA in this study was defined as a 50% increase in high intensity behaviours detected by behaviour analysing software, rather than direct activity measurements, it may be that less incremental activity is needed during the light phase, when animals are less active. Mice provided with 2, 3 or 6 meals a day at 12, 8 or 4 h intervals were able to predict the delivery of up to 4 meals per day, but, significantly, had only limited ability to predict meals on an 18 h (*i.e.*, non-circadian) schedule. This implies that the predictability of a meal may be determined by its timing relative to the circadian cycle rather than the interval between meals, with consequent effects on activity levels. The use of behavioural software to measure FAA also raises the question of which behaviours are contributing to FAA—increases in locomotor activity or an accumulation of micro-behaviours such as rearing or grooming, which may provide insight into disorders such as anxiety.

The elevated activity levels associated with food availability in a scheduled access paradigm will contribute to overall energy expenditure, so that the duration of FAA on a given day, but more importantly in the long term, is likely to impact on energy homeostasis. By contrast, mice that were intermittently and randomly food restricted by 50% or that had food completely removed showed significant reductions in physical activity both on days when the restriction was applied but also on subsequent days [56]. This would suggest that intermittent feeding schedules which prevent the reliable prediction of food availability may reduce overall energy expenditure, perhaps in compensation for reduced energy intake. However, if this adaptation is maintained during periods when food is again available it may result in positive energy balance and weight gain.

In a feeding schedule where palatable food (Oreo cookies) was given *ad lib.* for short periods of time (*i.e.*, 3 days out of 12) in order to create a background of restriction and refeeding similar to what is observed when dieting is undertaken (and fails) in humans, there was no effect in the standard anxiety tests, open field or elevated plus maze [57]. This manipulation, a 12-day period during which rats had periods of restriction followed by 7 days *ad lib.* access to chow, 3 of which also provided additional palatable food, presumably makes it difficult for animals to precisely foresee access to the palatable diet. Although a history of intermittent feeding on this schedule caused a generalised trend towards increased anhedonia, central changes in serotonin and dopamine were observed only when a history of caloric restriction was accompanied by intermittent access to cookies and not by *ad lib.* chow or daily access to palatable cookies [57]. This suggests that FAA is not the only behaviour associated with intermittent feeding schedules, but that traits such as depression and anxiety, which are important for human eating disorders, should also be considered. In a feeding schedule where rats were fed chow for 5 days followed by palatable diet for 2 days, binge eating developed during the palatable diet period, along with compulsive-like feeding characteristics [9]. Anxiety testing revealed complex outcomes with reduced exploratory behaviour in the open field test and reduced plasma corticosterone response to acute stress, but no effects on novel object exploration or in the elevated plus maze [9].

### 3.3. Summary

In combination, these studies suggest that intermittent access may be less anxiogenic than predictable access to palatable food, however, it is likely that the different timings and design of unpredictable feeding schedules will impact on their metabolic and anxiolytic properties. Further research and optimisation of these timings may allow them to be applied in a manner which could positively impact on the anxiety-related characteristics of eating disorders as well as other mental health conditions. Currently, therapeutic strategies to tackle binge eating disorder, for example, recommend normalisation of eating patterns through adherence to regular meals and snacks.

## 4. Potential Mechanisms of Behaviours Induced by Intermittent Feeding

### 4.1. Circadian Regulators

Rhythms in biology are generally thought of as being underpinned by central mechanisms driven through the suprachiasmatic nucleus (SCN). The SCN is part of a central clock system located within the hypothalamus which under normal conditions uses light to generate and regulate daily cycles in behaviours such as sleeping and eating (for review [58]). Many behaviours and physiologies are associated with such circadian rhythms, and food-entrained circadian patterns are thought to be responsible for a number of the metabolic changes related to food anticipation. Rhythms generated by feeding have long been known to persist even after lesions of the SCN [59] highlighting that peripheral tissues may also have a timekeeping capacity and be able to respond to feedback from food intake in order to promote food associated behaviours such as FAA [60]. It has been suggested that food-entrained clocks allow rats to respond to two meals a day during the light phase, with FAA increased for the second meal, while the removal, or a shift in the timing, of the first meal, to eliminate this as an indicator of time until the second meal, did not affect the level of FAA preceding the second meal [61]. While the time of food administration relative to normal fluctuations in activity may go some way to account for the elevated level of activity seen at the second meal, it is unclear to what extent food entrained oscillators (FEO) are responsible for FAA and, within this conceptualised framework, what happens to these clocks when availability of food becomes more unpredictable. When food availability does not align with feeding signals from the central clock it is possible that the interval between feeding opportunities as well as hormonal changes post intake may contribute to feedback allowing adjustments to be made to feeding behaviours [29]. Genes responsible for mediating circadian rhythms appear to respond predominantly to availability of food in general rather than palatable supplementary treats. Key regulators involved in the maintenance of the central clock include period 1 (PER1) and period 2 (PER 2) and disruption of the expression of such genes can impact on energy balance and FAA [62]. PER1 positive cells increase in the SCN, dorsomedial hypothalamus (DMH) and nucleus accumbens in food-restricted rats following a 2 h chow meal and also following a supplementary meal of chocolate when chow is available *ad lib*. However, increased PER1 positive cell numbers in the arcuate nucleus (ARC) and perifornical area are seen only with a restricted feeding schedule, while cells of the prefrontal cortex respond only to chocolate [36]. PER2 expression was unaltered by the administration of supplementary chocolate Ensure Plus for 2 h in addition to *ad lib.* chow but there were indicators of central effects of supplementary treats as numbers of Fos positive cells were increased in both the hypothalamus and the forebrain [38]. When Ensure Plus was fed for 2 h to rats that were otherwise completely food deprived, both PER2 and c-Fos were activated compared with *ad lib*. fed chow controls. Under disruptive light conditions, food can be used to reset circadian rhythms since mice fed or fasted on alternate days were able to maintain normal circadian expression in clock genes regardless of manipulation of the light/dark cycle to 8 h (*i.e.*, 3 L:D cycles each 24 h) [63].

While hypothalamic clocks primarily in the SCN are clearly factors in the regulation of feeding patterns, other regions of the brain, for example, traditional feeding centers such as the arcuate nucleus (ARC) are likely to play a role, as well as interacting with the SCN to generate a feedback loop [64]. There is also evidence to support interactions between circadian and reward pathways which may be influenced by food composition and the timing of food availability [65]. The involvement of the central nervous system in regulating feeding behaviours such as FAA is clearly not dependent on a single area but on the interaction of numerous nuclei involved in various elements of feeding and reward.

### 4.2. Peripheral Hormones and Metabolites

To date, a critical neuroanatomical/neurochemical basis for food related rhythms and behaviours has proved elusive. While the anticipation of, and response to, food is thought to be regulated by multiple signal systems distributed throughout the body, which may be modulated by peripheral hormones such as leptin and ghrelin [66], these hormones and others produced by peripheral tissues in response to, or in anticipation of, feeding are sensed by the ARC and hence may also contribute to regulation of food intake and related behaviours at the level of the hypothalamus [67]. Leptin had been suggested to play a role in the modulation of hyperactivity following food restriction in both humans and rodents [68] but does not appear to be required for FAA [69,70]. When accustomed to a scheduled 2 h binge feed of palatable food, rats have increased circulating ghrelin at the time of expected feeding [33]. Ghrelin has also been associated with reward pathways as well as with anxiety and depression [71] and it is possible that peripheral hormones are contributing to the effects of feeding regimes on mood and behaviour. Despite this, examination of mouse lines suggests that FAA is not dependent upon ghrelin [69].

Restricted access to food may be a stressor, and in a standard feeding schedule where calorically-restricted rats expect limited access to a palatable food or chow they have significantly elevated plasma corticosterone [33]. In contrast, rats with 2 weeks prior exposure to sucrose solution showed suppression of the HPA axis with reduced corticosterone in response to restraint stress up to 3 weeks after the termination of the sucrose feeding regime, suggestive of lasting modulation of the stress/reward circuits [72]. Within the generic paradigm of feeding for only 2 h per day, rats fed at irregular intervals had elevated circulating corticosterone compared to those fed a time-fixed meal in the light phase, regardless of the intermeal interval [30]. Consequently, interactions between the HPA axis and feeding regime appear likely to contribute to activity- or anxiety-related phenotypes such as those described in relation to eating disorders.

### 4.3. Energy Balance and Reward Pathways

Both FAA and anxiety behaviours induced by palatable meals and/or intermittent feeding could be regulated by homeostatic feeding pathways or central reward systems. However, establishing the mechanisms behind intermittent feeding and reward of palatable meals is complicated by the feeding schedules themselves which may enforce an element of food restriction and negative energy balance that also directly impacts on energy balance and reward systems. Activation of pathways regulating reward and energy homeostasis is suggested by observations made with a 2 h restricted feeding protocol. Here, c-Fos positive cells were increased in the ARC and DMH 25 h after the start of the last meal regardless of whether the meal was predictable or unpredictable [30]. In addition, c-Fos induction in the hypothalamus was also observed in anticipation of food being made available in a similar 2 h feeding schedule [73]. Long term neuronal plasticity, as measured by FosB positive cells, was also elevated for up to 3 weeks following brief daily exposure to palatable sucrose solution for 14 days [72].

How intermittent feeding schedules can mediate energy balance and behaviour is not well understood. However, the potential role of the hypothalamus and feeding pathways was highlighted in similar studies where gene expression was analysed prior to anticipated palatable food availability with chow available *ad lib*. Rats fed palatable supplements for 2 h a day in addition to chow responded differently to different solid or liquid palatable foods. Rats with 2 h access to sucrose had reduced ARC expression of the homeostatic neuropeptides, cocaine and amphetamine-regulated transcript (CART) and proopiomelanocortin (POMC), and of suppressor of cytokine signalling-3 (SOCS3), compared to rats given *ad lib*. access to sucrose solution. A similar relationship was not observed when high fat pellets were schedule-fed, although neuropeptide Y (NPY) was upregulated by scheduled feeding of a 60% high fat pellet. Schedule-fed liquid chocolate Ensure reduced leptin receptor and SOCS3 expression but not that of the energy balance neuropeptides [28]. By comparison, the same schedules and dietary manipulations with mice resulted in elevation of NPY in schedule-fed animals with all the palatable diets (sucrose, high fat and Ensure) while schedule feeding the high fat and Ensure diets also suppressed CART and SOCS3, and increased leptin receptor gene expression [28]. Both studies by Bake *et al.* [28] quantified gene expression immediately prior to the anticipated access to palatable food. It would be interesting to correlate these gene expression changes with FAA, and NPY may be of particular interest since it was affected by scheduled feeding of all the palatable diets offered. Significantly, NPY Y1 receptor deficient mice had lower levels of FAA in anticipation of food [74] and while NPY may not be essential for FAA [69], this suggests that NPY acting via the Y1 receptor may play a role in the predictability of meals, and the regulation of FAA and related behaviours which also impact on feeding and reward pathways. The involvement of these genes in feeding behaviours when FAA is prevented by irregular feeding schedules and the significance of this for energy balance requires further investigation.

The hypothalamus may also play a role in regulating the behavioural response to feeding times by integrating signalling from peripheral hormones such as ghrelin. In an electrophysiological study, rats were held on a randomised single meal feeding schedule where the approaching meal, fed at a different time of the dark active phase, was signalled by a light cue [75]. The light cue stimulated both FAA and increased neural activity in the medial hypothalamus. Significantly, exogenous ghrelin administration also increased the firing of neurons in the DMH and ventromedial hypothalamus (VMH) and responses were highly correlated with those following cue-induced FAA. This would suggest that ghrelin signalling in the DMH/VMH region of the brain is important in the regulation of food anticipation, even if it is not essential [69]. The conclusion that ghrelin has a substantive role in food anticipation is supported by detailed transgenic and pharmacological manipulation studies using both mice and rats [76], and a number of other studies indicating that ghrelin receptor signalling is required to augment FAA [77,78]. Functional dopaminergic [79], serotonergic [80] or melanocortin-3 receptor dependent signalling systems [81,82] may also be required.

In terms of reward pathways, animals fed a palatable high fat-high sugar diet had suppressed expression of µ opioid receptors in the brainstem (nucleus of the solitary tract) and this was true both in rats fed the palatable diet option *ad lib*. or in a 2 h period following restriction to induce bingeing [83]. µ opioid receptor knock out mice had similar intake to wild types on a restricted schedule but showed suppression of FAA prior to scheduled feeding [84]. This would suggest that regulation of the opioid pathway, at least at the level of brainstem receptor expression, is mediated through diet composition rather than feeding schedule and may be associated with feeding behaviours such as activity. 

## 5. Discussion

This review aimed to integrate a varied and dispersed literature derived from imposed disrupted feeding regimes. Research in this area could have implications for our understanding of mood and eating disorders and their treatment. The increased impulsivity associated with obesity, and probably with binge eating disorder also [85], as well as the positive correlation between BMI and the ability to predict food reward [86] illustrates the relationship between mood, the predictably of food and overall consumption and energy balance. This is particularly evident in a study where fMRI identified increased activation of brain regions associated with reward during food anticipation in obese individuals, while central effects related to actual consumption were dampened in these individuals [87]. This behavioural inhibition and ability to predict feeding opportunities combined with the anxiety associated with eating disorders, as well as addictions [18,88], emphasises the wide potential impact of strategies to regulate behaviour and anxiety.

Animals tend to binge and display clear FAA that may be combined with disruption of circadian activity when palatable food is provided in a predictable manner alongside unrestricted access to chow. Animals are likely to experience anxiolytic symptoms as a result of such schedules but opposing anxiogenic effects during withdrawal. On the other hand, irregular feeding has a less robust effect on anxiety which may reflect reduced ability to predict the meal and to display FAA. Candidate molecules and neuroanatomical substrates from circadian, reward and energy regulating pathways and circuits have all been implicated in mediating the effects of meal timing but interpretation of results from these studies is complicated by the confounding component of food restriction in the feeding schedules with the consequence that the strongest contenders are those which directly affect FAA such as NPY.

The range of feeding studies discussed here includes regimes of starvation, restriction, refeeding and intermittent access, but it is worth noting that in the different studies these phrases are sometimes used interchangeably depending on the focus of the respective research group. Similarly, these feeding schedules have been used in a range of studies to address research outcomes that do not necessarily include the effect of the schedule on the animal itself in terms of food intake or feeding-related behaviour. In addition, there is a paucity of information on behavioural consequences other than FAA during such regimes, and effects on later feeding behaviour and specifically other mood-related behaviours such as anxiety. Where the feeding schedule was the primary manipulation, the main focus of current literature has been on effects following withdrawal of feeding schedules of palatable food, and subsequent effects on intake, rather than the potential of feeding schedules to mediate metabolic or mood disorders associated with eating disorders. Studies of feeding behaviour following the termination of intermittent or irregular feeding patterns do, however, highlight the importance of dietary history as well as current eating regime on mood and behaviour outcomes. Our aim in integrating this varied body of research was to highlight that apparently simple manipulations of food availability have the potential to impact on more widely relevant behaviours with application in the study of eating disorders.

While feeding behaviour and FAA are clearly influenced by diet availability and the predictability of meals, effects on other food-related behaviours such as anxiety are less well reported. Mental health conditions are closely associated with eating disorders and it is not surprising, therefore, that disrupting feeding patterns in animal models may impact on behaviour. More emphasis should be placed on monitoring such behaviours in situations where food intake is manipulated for other reasons. A better understanding of the behavioural consequences of disruption to normal eating patterns may be important in improving both clinical diagnostic criteria and therapies for eating disorders such as binge eating disorder which is characterised by intake of irregular high calorie meals. A more detailed characterisation of pre-clinical models may further our understanding of how such conditions impact on people’s mood, behaviour and daily lives.

Changes in availability of the main dietary energy source may have a larger impact on circadian and metabolic rhythms, and FAA, than palatable treat foods [38,39], but both eating events have the potential to impact on the reward value of food as well as mood. An increased awareness of how individuals decide when to consume most of their energy intake, and the factors influencing these decisions, may help to maximise beneficial effects of meal timing on mood, behaviour and long term energy homeostasis. Future studies are needed to establish the potential role of food in modulating feeding behaviour, activity and anxiety, not only as a predictor of disease, but also as a treatment. There may be opportunities to use feeding schedules in the treatment of eating disorders, beyond current recommendations to keep track of eating and adopt regular meal and snack eating patterns, but wider implications for mood, behaviour and energy regulation need to be more clearly understood.

## 6. Conclusions

It is apparent that there has been only limited research into the direct effects of feeding schedules on behaviour. Consequently, establishing the potential for intermittent feeding schedules to generate behavioural responses beyond classical feeding and activity responses will require further investigation. The combination of changes in energy intake and anxiety-associated behaviours may link animal models of intermittent feeding with human eating disorders, and allow the development of models to promote our understanding of underlying endocrine and neurobiological mechanisms, and facilitate the identification of therapeutic targets. The relationship between food predictability and the anxiolytic/anxiogenic properties of a particular diet suggests that habitual dietary manipulations may be useful as indicators of eating disorders and traditional addictions, whereas dietary strategies may have a therapeutic benefit in addressing behaviours associated with such conditions.
